# Serum uric acid to albumin ratio and C-reactive protein as predictive biomarkers for chronic total occlusion and coronary collateral circulation quality

**DOI:** 10.1515/med-2025-1299

**Published:** 2025-11-15

**Authors:** Yufei Zhao, Jianming Zhang, ErLi Yang, Juan Wu, Shouye Dong

**Affiliations:** Cardiovascular Department for Gerontism, The Second Affiliated Hospital of Anhui Medical University, Hefei, Anhui, China; Department of Intervention, Yingshang County Hospital of Traditional Chinese Medicine, Yingshang, Fuyang, Anhui, China; Department of Cardiology, Yingshang County Hospital of Traditional Chinese Medicine, No. 6, Nanwei San Road, Shencheng Town, Yingshang County, Fuyang City, Anhui Province, China

**Keywords:** chronic total occlusion of coronary arteries, coronary collateral circulation, uric acid/albumin ratio, C-reactive protein

## Abstract

**Background:**

Coronary collateral circulation (CCC) plays a vital role in preserving myocardial perfusion in patients with chronic total occlusion (CTO). Reliable biomarkers for evaluating CCC are needed. This study aimed to investigate the combined diagnostic value of serum uric acid-to-albumin ratio (UAR) and C-reactive protein (CRP) in assessing the CCC status.

**Methods:**

We enrolled 220 patients undergoing coronary angiography and categorized them into three groups: CTO (*n* = 80), coronary heart disease (CHD, *n* = 100), and controls (*n* = 40). Serum UAR and CRP levels were measured. Logistic regression and receiver operating characteristic curve analyses were performed to determine associations with CCC quality.

**Results:**

UAR and CRP levels were significantly elevated in the CTO group compared to CHD and control groups. Among CTO patients, those with well-developed CCC had significantly lower UAR and CRP levels. Both biomarkers were identified as independent predictors of the CCC status. Combined use of CRP and UAR improved diagnostic specificity to 91.1%.

**Conclusion:**

This is the first study to demonstrate the diagnostic utility of combining UAR and CRP for evaluating CCC in CTO patients. The dual-marker approach enhances diagnostic accuracy and may support more precise clinical decision-making in coronary artery disease.

## 1 Introduction

Coronary artery disease (CAD) remains a leading cause of cardiovascular mortality worldwide, characterized by atherosclerotic plaque formation and progressive arterial stenosis [1,2,3]. With population aging and lifestyle changes, the incidence of CAD continues to rise, posing substantial challenges to global healthcare systems [4,5,6]. Chronic total occlusion (CTO), the most advanced form of CAD, involves complete coronary artery obstruction for more than three months [7,8,9]. CTO significantly impairs myocardial perfusion, resulting in ischemia or infarction and adversely affecting patient quality of life and prognosis [10,11,12]. Despite advancements in interventional cardiology, CTO remains a clinical challenge, with low revascularization success, high restenosis rates, and variable outcomes [13,14,15].

Coronary collateral circulation (CCC) serves as an adaptive mechanism that maintains myocardial perfusion in the setting of coronary artery obstruction. Well-developed CCC can reduce ischemic burden, preserve myocardial function, and improve long-term outcomes [16,17,18]. The extent of CCC development is closely associated with survival after myocardial infarction, recovery of cardiac function, and overall prognosis [19,20]. Clinically, CCC evaluation is essential for guiding CTO management and predicting therapeutic response [21,22]. However, its formation is influenced by various factors, including genetics, metabolic status, and comorbid conditions [23], highlighting the need for reliable biomarkers to assess CCC status.

Recent advances in biomedical research have promoted the use of serum biomarkers in evaluating CTO and CCC [24]. Uric acid (UA), albumin (Alb), and C-reactive protein (CRP) have been studied for their pathophysiological relevance in cardiovascular disease [25,26]. UA is a marker of oxidative stress, which contributes to CAD progression [27,28]; decreased albumin reflects systemic inflammation and malnutrition [29], and elevated CRP indicates acute inflammatory activity [30]. Prior studies have shown that elevated UA levels are significantly associated with impaired CCC in patients with acute coronary syndrome, stable CAD, and CTO [3,31,32]. The UA-to-albumin ratio (UAR) has emerged as a composite biomarker integrating metabolic and inflammatory status [33]. However, the combined diagnostic value of UAR and CRP in assessing CCC remains underexplored.

Therefore, this study aimed to evaluate, for the first time, the combined utility of UAR and CRP in assessing CCC development in CTO patients. Based on clinical and biochemical data from 220 individuals undergoing coronary angiography, we investigated their potential as biomarkers to improve diagnostic accuracy and support personalized treatment strategies.

## 2 Materials and methods

### 2.1 Research subject

**Figure 1:**
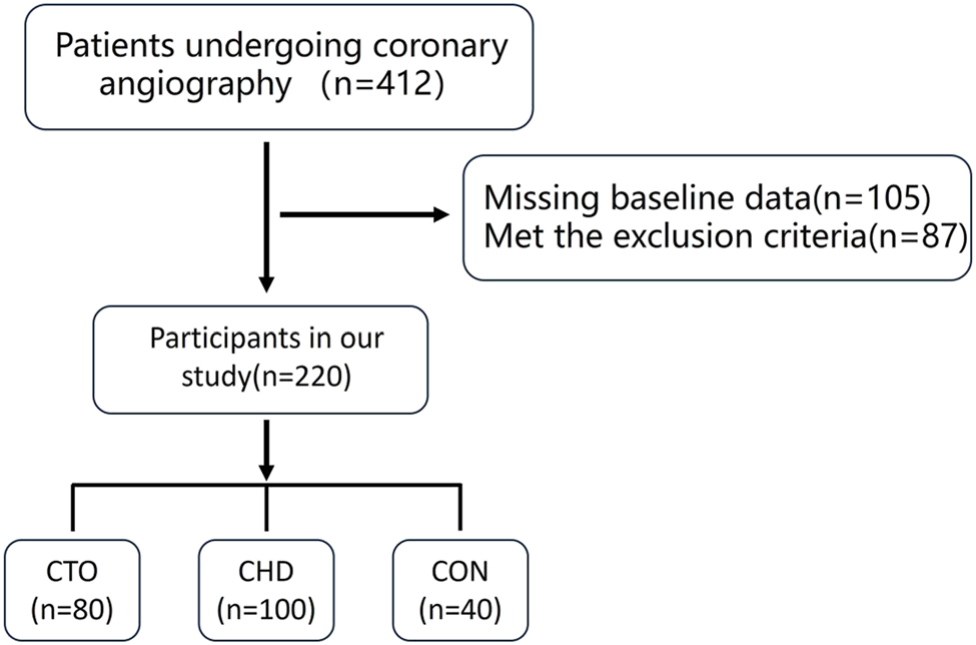
Flowchart of the participant inclusion and exclusion processes. Note: CTO: chronic total occlusion; CHD: coronary heart disease (stenosis); CON: control.

This study included patients who underwent coronary angiography at the First Affiliated Hospital of Jinzhou Medical University from January 2019 to December 2022 (Figure 1). Inclusion criteria were: age 18 years or older and complete baseline blood biochemistry data (including UA, albumin, CRP, triglycerides, etc.). The patients were divided into three groups: the CTO group (80 cases), where coronary angiography revealed at least one completely occluded vessel with an occlusion duration of more than three months; the coronary heart disease (CHD) group (100 cases), where angiography showed at least one vessel with more than 50% stenosis; and the normal control group (CON) (40 cases), where no significant stenosis was observed on angiography. The CHD group was included as an intermediate pathological state between normal coronary arteries and CTO, which helps to clarify the dynamic changes in UAR and CRP across different stages of CAD. This three-group comparison enhances the interpretive value of the study and supports the potential of UAR and CRP as stratified diagnostic biomarkers. Exclusion criteria included structural heart disease, malignant tumors, rheumatic or autoimmune diseases, liver or renal dysfunction, recent use of immunosuppressive drugs, neurodegenerative diseases, and recent acute inflammation or infection.

All sensitive personal information (e.g., name, ID number) was anonymized and replaced with unique identification codes to ensure patient privacy. Data were stored in the hospital’s secure database, accessible only to core research team members, ensuring data security. The analysis results did not contain any personally identifiable information. This study was approved by the Clinical Ethics Committee of The Second Affiliated Hospital of Anhui Medical University (approved number: SL-YX(YS)2023-SZR 092), and informed consent was obtained from all participants. The study strictly adhered to the principles outlined in the Declaration of Helsinki [34].

### 2.2 Research methods

Patient information, including name, gender, age, height, weight, body mass index (BMI), and medical history (hypertension, diabetes, stroke, smoking history), was collected. Within 24 hours of admission, fasting venous blood samples were obtained in the early morning and analyzed using a whole blood automated analyzer to measure white blood cell count (WBC), hemoglobin (Hb), and platelet levels. Additionally, an automated chemiluminescence immunoassay analyzer was used to measure alanine aminotransferase (ALT), aspartate aminotransferase (AST), blood urea nitrogen (BUN), glucose (Glu), UA, creatinine (CR), CRP, total cholesterol (TC), triglycerides (TG), total protein (TP), albumin (ALB), albumin/globulin ratio, and UAR. Coronary angiography was performed using the Judkins method, and collateral circulation was classified into Rentrop 0 to 3 levels based on the coronary angiogram findings [35]: Grade 0 indicates no visible collateral vessels, Grade 1 indicates some filling of the side branch, Grade 2 represents partial filling of the epicardial vessel in CTO, and Grade 3 indicates complete filling of the epicardial vessel.

### 2.3 Statistical analysis

In this study, all statistical analyses were performed using SPSS 22.0 software. Continuous variables with a normal distribution were expressed as mean ± standard deviation and compared between groups using *t*-tests or analysis of variance. Continuous variables with a non-normal distribution were expressed as the median and interquartile range, with group comparisons performed using the Mann–Whitney *U* test or Kruskal–Wallis test. Categorical variables were described as proportions, and group comparisons were conducted using the chi-square test (*χ* ^2^ test) or Fisher’s exact test. Normality tests were performed for all continuous variables, and Bonferroni correction was applied for multiple comparisons, with *P* < 0.05 considered statistically significant [36].

To explore the impact of CRP and UAR on CCC formation in CTO patients, binary logistic regression analysis was conducted to assess the independent predictive value of these biomarkers based on the correlation analysis. In the study, 80 CTO patients were divided into a low collateral circulation group (Rentrop grades 0–1) and a high collateral circulation group (Rentrop grades 2–3) according to the Rentrop grading. Collateral circulation formation (0 = low collateral circulation, 1 = high collateral circulation) was set as the dependent variable, while CRP, UA, and UAR were the primary independent variables. Variables with statistical significance (*P* < 0.05) were initially identified through univariate analysis and directly included in the regression model. The regression analysis employed the maximum likelihood estimation method, with results presented as odds ratios (OR) and their 95% confidence intervals (CI). The model’s goodness-of-fit was evaluated using the Hosmer–Lemeshow test, and model independence was validated through residual analysis and standardized coefficients to ensure the robustness of the results.

## 3 Results

### 3.1 Elevated biochemical markers in patients with CTO: Implications for pathophysiology and management

Baseline characteristics were compared among the CHD, CTO, and CON groups, as shown in Table 1. A total of 220 patients were included in this study, with 114 males (51.81%) and 106 females (48.19%), and a mean age of 63.35 ± 10.94 years. There were no statistically significant differences in age, gender, BMI, or histories of hypertension, diabetes, stroke, and smoking across the three groups, indicating comparable baseline characteristics.

**Table 1:** Baseline characteristics comparison among normal, coronary heart disease, and chronic total occlusion groups

Project	CON group (*n* = 40)	CHD group (*n* = 100)	CTO group (*n* = 80)	*H*/*χ* ^2^ value
Age (years)	66.00 (56.75, 69.00)	65.50 (57.25, 71.75)	68.00 (55.25, 74.00)	1.706
BMI (kg/m^2^)	22.92 (20.63, 26.29)	23.30 (20.72, 26.10)	22.77 (20.30, 25.29)	1.414
Male patients/cases (%)	19	51	52	4.779
History of hypertension/cases (%)	22	58	54	2.402
History of diabetes/cases (%)	12	21	28	4.473
History of stroke/cases (%)	1	12	6	2.826
Smoking history/cases (%)	6	24	27	2.232
WBC (*10^9^)	6.39 ± 2.12	6.58 ± 2.04	6.43 ± 1.58	0.211
Hb (g/L)	131.00 (126.00, 137.50)	132.00 (124.25, 140.00)	130.00 (121.00, 140.00)	2.187
PLT (*10^9^)	239.00 (202.25, 300.25)	221.00 (175.25, 264.50)	210.50 (174.25, 264.50)	5.352
ALT (U/L)	38.97 ± 49.57	36.88 ± 72.88	44.06 ± 80.99	0.221
AST (U/L)	54.25 ± 78.94	38.86 ± 72.14	50.46 ± 67.30	0.910
BUN (mmol/L)	7.16 ± 3.06	7.70 ± 5.00	8.08 ± 3.43	0.659
Glu (mmol/L)	5.06 (4.57, 5.89)	5.04 (4.47, 5.59)^*^	5.43 (4.72, 6.81)^*^	6.693
UA (µmol/L)	254.00 (192.50, 287.50)	291.50 (242.50, 329.75)^*^	328.50 (277.39, 390.00)^*#^	30.182
CR (µmol/L)	59.00 (52.25, 67.00)	66.00 (59.25, 72.00)^*^	67.00 (59.25, 72.00)^*^	15.088
CRP (mg/L)	2.98 ± 2.17	7.83 ± 9.90^*^	8.08 ± 3.43^*^	-3.019
TC (mmol/L)	3.72 ± 0.92	4.12 ± 1.05	4.32 ± 1.64	2.953
TG (mmol/L)	1.51 ± 0.89	1.58 ± 0.75	2.28 ± 2.12^*#^	6.505
TP (g/L)	63.95 (60.33, 68.88)	64.15 (59.33, 68.70)	65.10 (60.08, 69.05)	0.768
ALB (g/L)	38.60 (35.90, 40.95)	35.88 (36.03, 41.75)	40.15 (36.35, 42.40)	2.895
Albumin/globulin ratio	1.52 (1.30, 7.12)	1.51 (1.33, 1.78)	1.52 (1.36, 1.67)	2.312
UAR	6.57 (5.12, 8.03)	7.55 (6.20, 8.75)^*^	8.30 (7.55, 9.97)^*#^	20.826

Note: CTO: chronic total occlusion; CHD: coronary heart disease; CON: control, BMI: body mass index, WBC: white blood cell, PLT: platelet, ALT: alanine aminotransferase, AST: aspartate aminotransferase, BUN: blood urea nitrogen, Glu: glucose, UA: uric acid, CR: creatinine, CRP: C-reactive protein, TC: total cholesterol, TG: triglyceride, TP: total protein, ALB: albumin, UAR: uric acid-to-albumin ratio,*p < 0.05 vs CON group; #*p* < 0.05 vs CHD group.

**Figure 2:**
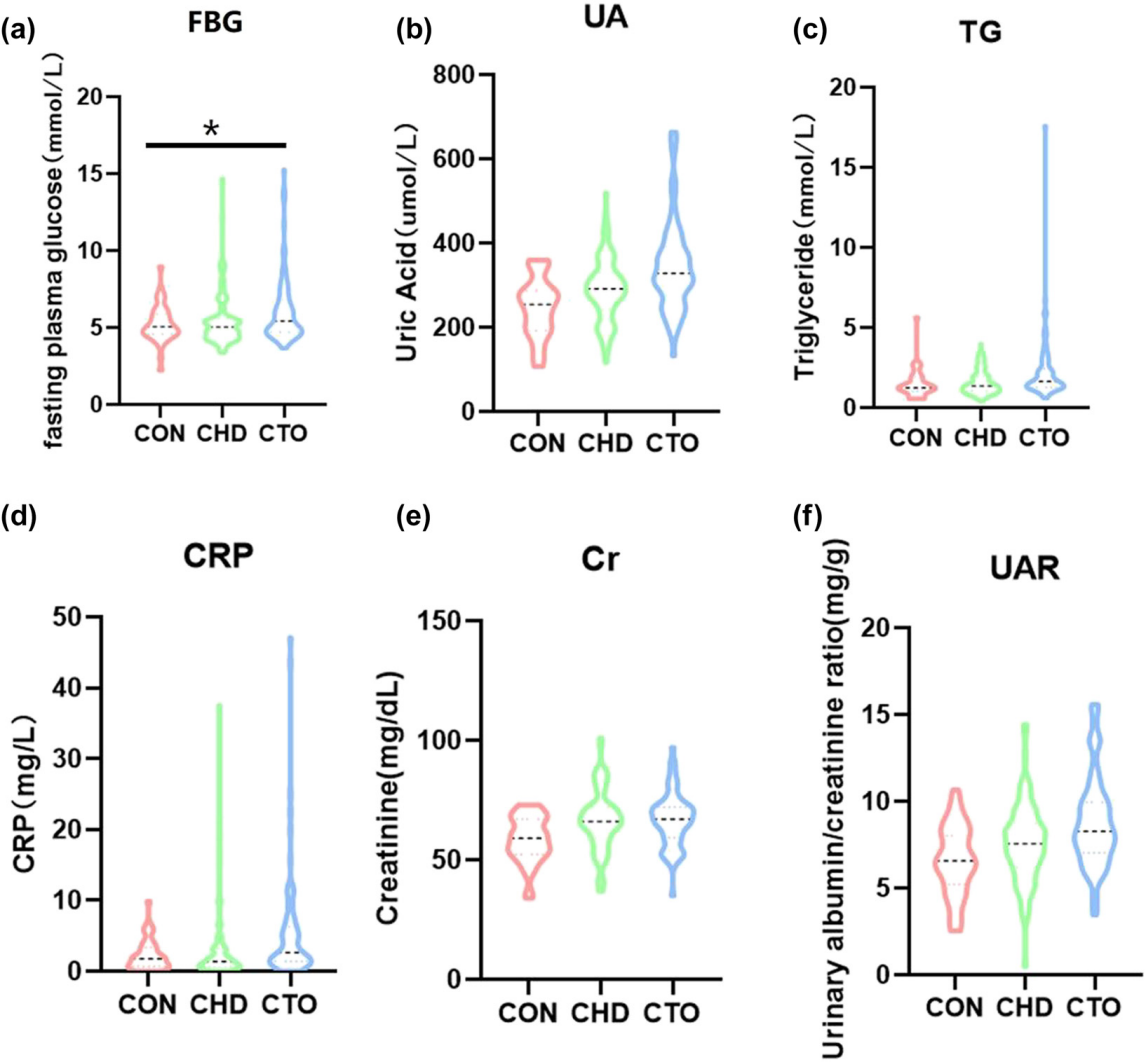
Violin plot illustrating variances in baseline data metrics. Note: Comparison of pre-test results between the CTO (chronic total occlusion) group, CHD (coronary heart disease) group, and CON (control) group for (a) fasting blood glucose, (b) uric acid, (c) triglycerides, (d) C-reactive protein, (e) creatinine, and (f) urinary albumin-to-creatinine ratio. Statistical significance: *p < 0.05, **p < 0.01, ***p < 0.001.

No significant differences were observed in several biochemical parameters, including WBC, Hb, PLT, ALT, AST, BUN, TC, TP, ALB, and the albumin/globulin ratio. The glucose level in the CTO group (5.43 mmol/L) was significantly higher than in the CON group (5.06 mmol/L, *p* = 0.03) (Figure 2a). The UA level in the CTO group (328.50 μmol/L) was significantly higher than in the CHD (291.50 μmol/L) and CON (254.00 μmol/L) groups (*p* < 0.001) (Figure 2b). Triglyceride levels were higher in the CTO group (2.28 mmol/L) than in the CHD and CON groups (*p* < 0.01) (Figure 2c). CRP levels were significantly elevated in the CTO and CHD groups compared to the CON group (*p* < 0.01) (Figure 2d). Creatinine levels in the CTO and CHD groups were higher than in the CON group (*p* < 0.01) (Figure 2e). UAR levels were also significantly increased in the CTO group (8.30) compared to the CHD group (7.55) and the CON group (6.57) (*p* < 0.001) (Figure 2f).

### 3.2 Association of CRP and UAR levels with collateral circulation formation in CTO patients

Eighty CTO patients were categorized into the low collateral circulation group (Rentrop grades 0–1) and high collateral circulation group (Rentrop grades 2–3) based on the Rentrop classification (Table 2). There were no statistically significant differences in age, BMI, sex ratio, or histories of hypertension, diabetes, stroke, and smoking between the two groups (*p* > 0.05), indicating comparable baseline characteristics. No significant differences between the two groups were observed in WBC, Hb, PLT, ALT, AST, BUN, Glu, TC, TG, TP, or ALB levels. The high collateral circulation group had lower UA levels (303.50 μmol/L vs 344.00 μmol/L, *p* = 0.012), lower CRP levels (*p* = 0.001), and lower UAR values (7.22 vs 9.50, *p* = 0.001) compared to the low collateral group. Binary logistic regression analysis was performed on variables with significant differences. CRP was identified as an independent predictor of collateral circulation (OR = 0.717, 95% CI: 0.582–0.882, *p* = 0.002), as was UAR (OR = 0.550, 95% CI: 0.317–0.953, *p* = 0.033). UA was insignificant in the regression model (*p* = 0.317) (Table 3).

**Table 2:** Baseline data comparison of CTO patients with different levels of coronary collateral circulation

Project	Lower limb circulation group (*n* = 46)	Upper limb circulation group (*n* = 34)	*t*/*F* value	*P* value
Age (years)	65.02 ± 13.54	64.56 ± 10.06	0.175	0.861
BMI/(kg/m^2^)	22.51 (20.02, 26.51)	23.31 (20.13, 25.66)	0.168	0.851
Male patients/cases (%)	28 (60.87)	24 (70.59)	0.812	0.368
History of hypertension/case (%)	31 (67.39)	23 (67.64)	0.001	0.981
History of diabetes/cases (%)	19 (41.30)	9 (26.47)	1.891	0.169
History of stroke/cases (%)	3 (9.67)	3 (8.82)	0.149	0.699
Smoking history/cases (%)	18	9	1.401	0.236
WBC (*10^9^)	5.79 (4.85, 7.40)	6.87 (5.55, 8.03)	1.830	0.067
Hb (g/L)	129.00 (120.00, 135.00)	133.00 (122.00, 139.00)	1.694	0.090
PLT (*10^9^)	209.00 (176.50, 258.25)	512.50 (164.50, 259.00)	0.214	0.830
ALT (U/L)	44.41 ± 97.03	43.59 ± 53.53	0.049	0.961
AST (U/L)	50.70 ± 67.76	50.15 ± 67.69	0.360	0.972
BUN (mmol/L)	7.09 (5.49, 9.41)	7.95 (5.26, 10.13)	00.341	0.733
Glu (mmol/L)	6.01 ± 2.28	6.27 ± 2.16	0.515	0.608
UA (µmol/L)	344.00 (305.00, 393.00)	303.50 (238.50, 375.25)	2.501	0.012
CR (µmol/L)	65.50 (57.00, 71.25)	68.00 (64.00, 74.25)	1.300	0.193
CRP (mg/L)	8.04 ± 9.85	2.229 ± 2.07	3.347	0.001
TC (mmol/L)	4.13 ± 1.64	4.57 ± 1.61	1.121	0.230
TG (mmol/L)	2.63 ± 2.60	1.81 ± 1.04	1.946	0.056
TP (g/L)	67.10 (62.13, 69.80)	61.10 (65.43, 67.50)	1.956	0.050
ALB (g/L)	41.30 (37.10, 42.70)	38.60 (35.68, 41.90)	1.538	0.124
Albumin/globulin ratio	1.53 (1.38, 1.66)	1.49 (1.29, 1.69)	0.540	0.089
UAR	9.50 (9.11, 10.27)	7.22 (6.00, 8.74)	3.337	0.001

Note: CTO: chronic total occlusion, BMI: body mass index, WBC: white blood cell, PLT: platelet, ALT: alanine aminotransferase, AST: aspartate aminotransferase, BUN: blood urea nitrogen, Glu: glucose, UA: uric acid, CR: creatinine, CRP: C-reactive protein, TC: total cholesterol, TG: triglyceride, TP: total protein, ALB: albumin, And UAR: uric acid-to-albumin ratio.

**Table 3:** Binary logistic regression analysis of factors influencing collateral circulation in CTO patients

Project	CON group (*n* = 40)	CHD group (*n* = 100)	CTO group (*n* = 80)	*H*/χ^2^ value	*P* value
CRP	−0.559	0.106	9.886	0.717 (0.582–00.882)	0.002
UA	0.008	0.008	1.001	1.008 (0.993–1.023)	0.317
UAR	−0.559	0.281	4.548	00.550 (0.317–0.953)	0.033

Note: UA: uric acid, CRP: C-reactive protein, UAR: uric acid-to-albumin ratio, CTO: chronic total occlusion, CON: control group, and CHD: coronary heart disease.

### 3.3 Predictive values of serum CRP and UAR for collateral circulation in patients with CTO

**Figure 3:**
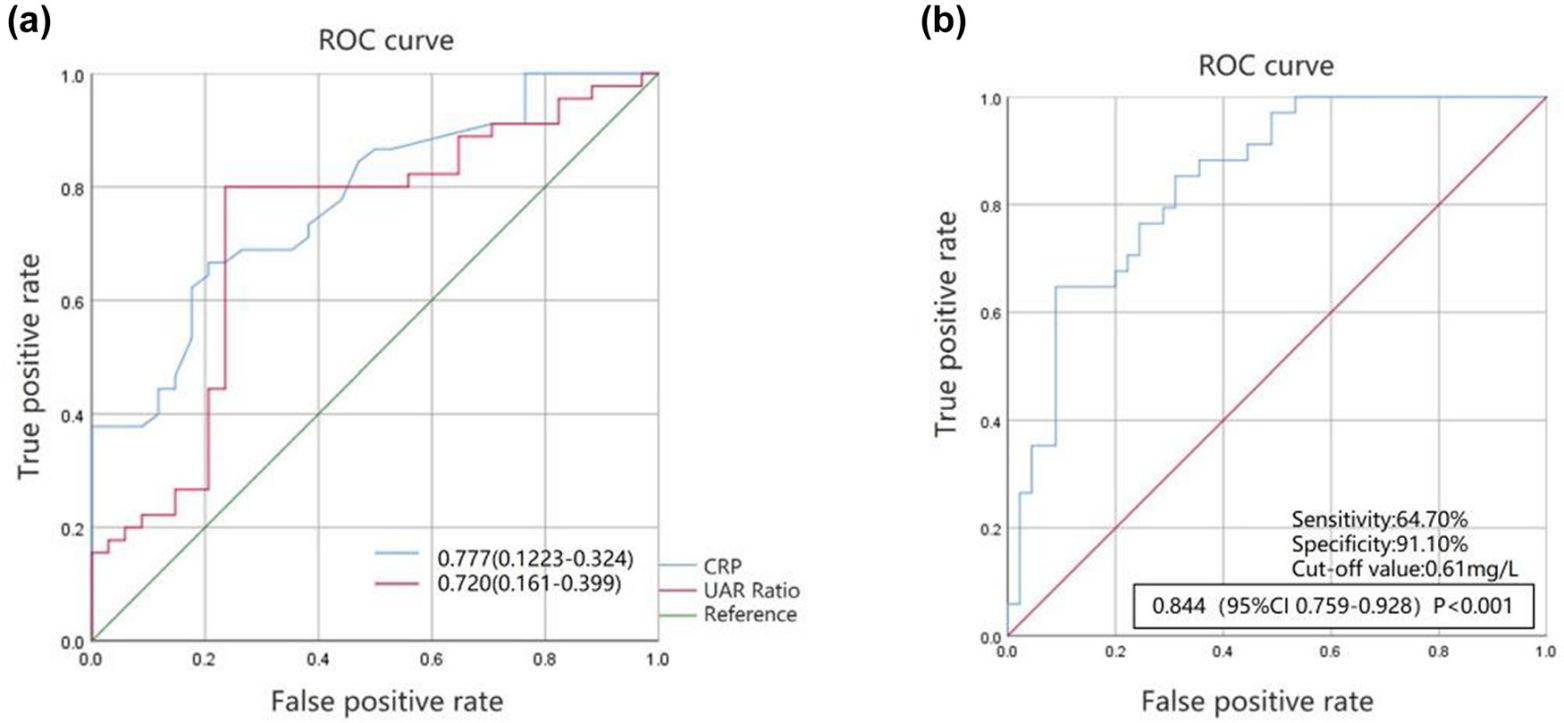
ROC curves of the logistic regression model. Note: (a) ROC curve for serum CRP and UAR in predicting the presence of CCC in CTO patients; (b) ROC curve for the combined prediction of CCC in CTO patients using serum CRP and UAR. CRP: C-reactive protein; CTO: chronic total occlusion; CHD: coronary heart disease (stenosis); CON: control; UAR: uric acid-to-albumin ratio.

Receiver operating characteristic (ROC) curves were constructed for CTO patients based on high collateral circulation. The results revealed that the area under the ROC curve for CRP was 0.777 (95% CI 0.223–0.324), with an optimal cutoff value of 3.19 mg/L, sensitivity of 66.70%, and specificity of 79.40%. The area under the ROC curve for UAR was 0.720 (95% CI 0.161–0.399), with an optimal cutoff value of 8.74, sensitivity of 80.00%, and specificity of 76.50% (Figure 3a). When both biomarkers were used for diagnosis, the combined area under the ROC curve was 0.844 (95% CI 0.759–0.928, *P* < 0.001), with an optimal cutoff value of 0.61 mg/L, sensitivity of 64.70%, and specificity of 91.10% (Figure 3b).

## 4 Discussion

In the research field of cardiovascular diseases, studies on serum UAR and CRP as risk factors have been increasing [37,38]. Previous studies have indicated that UA, an oxidative stress marker, is closely associated with various cardiovascular events [39,40]. Furthermore, as a negative acute-phase protein, albumin is widely reported to decrease under inflammatory conditions [41,42,43]. As a sensitive inflammation marker, CRP is crucial in predicting cardiovascular diseases [42,44,45]. In comparison to previous studies, our research, for the first time, establishes a connection between UAR and CCC in CTO patients, suggesting UAR as a novel biomarker for evaluating the collateral circulation status of CTO patients. It differs from prior literature that solely focused on UA or albumin as assessment indicators [37], providing a more comprehensive biochemical parameter analysis in our study.

Our study results demonstrate that UAR is significantly lower in the high collateral circulation group than in the low collateral circulation group, indicating that CTO patients with lower UAR may have better collateral circulation formation. The significant increase in UAR and CRP levels in the CTO group may reflect the combined effect of inflammation and oxidative stress in CTO patients. UA, as a marker of oxidative stress, may be elevated due to purine metabolism disorders, which could further exacerbate the progression of coronary atherosclerosis in CTO patients [26]. This finding is consistent with recent studies suggesting the dual role UA might play in cardiovascular health [46,47,48]. However, unlike other studies predominantly concentrating on the impact of UA at a single level [39], our research offers a new perspective by combining UA and albumin ratio to evaluate this relationship. Furthermore, our study further explores the connection between UAR and CTO and CCC formation, filling gaps in prior research.

In addition, CRP levels are closely associated with CTO and the formation of CCC. As a marker of the inflammatory response, elevated CRP may impair collateral vessel development by inhibiting endothelial cell function or enhancing vascular wall inflammation [49]. This finding is consistent with previous studies identifying CRP as a key inflammatory marker in cardiovascular disease [42,45,50]. While most existing research has focused on the relationship between CRP and cardiovascular event risk, our study further expands its potential clinical application in assessing collateral circulation in CTO patients [41,51,52]. Both CRP and UAR demonstrate good diagnostic value in predicting collateral vessel formation in CTO patients. Although CRP alone performs slightly better, its combined use significantly enhances diagnostic accuracy, improving sensitivity and specificity. Therefore, the combined measurement of CRP and UAR may offer a more reliable basis for evaluating CCC development in CTO patients, aiding in risk assessment and supporting individualized treatment strategies.

TG levels in CTO patients may aggravate coronary atherosclerosis and further impair collateral circulation formation, highlighting the role of lipid metabolism disorders in CTO pathophysiology [53]. Likewise, UAR was significantly higher in CTO patients, suggesting the combined influence of inflammation and metabolic dysfunction. As UAR reflects decreased albumin and elevated UA, impacting the disease state, its elevation may be clinically relevant [33]. Our findings show that combining UAR and CRP improves CTO and collateral circulation status diagnostic specificity. It offers a more precise tool for risk stratification and treatment planning. While prior studies have examined UAR and CRP individually as cardiovascular risk indicators, few have assessed their combined utility [54,55]. Our results suggest that their joint application may enhance clinical decision-making and support the broader use of these biomarkers in managing CTO, potentially improving diagnostic and prognostic outcomes.

This study identifies UAR and CRP as risk factors for the formation of CCC in patients with CTO, contributing to cardiovascular research and offering new tools for clinical risk assessment. These markers may aid in evaluating collateral circulation and guiding personalized treatment. However, limitations such as a small sample size and a single-center design may affect generalizability. UAR and CRP levels may also be influenced by metabolic disorders, chronic inflammation, or medications, highlighting the need to control for confounding factors in future research. Larger multicenter studies are needed to validate these findings and explore combined biomarker approaches to improve the diagnosis and treatment of CTO and CCC. Proteomic methods, such as those used in the MESA study [56], may help identify additional relevant biomarkers. The mechanisms by which UAR and CRP affect CCC development remain unclear. While hyperuricemia may be linked to hypoxia-induced purine metabolism in poorly perfused tissues, further studies are needed to clarify the molecular pathways. Future research should also examine the temporal dynamics of these biomarkers and their variability across disease stages and populations to support advances in precision cardiovascular medicine. Although CCC is typically assessed via invasive angiography at the time of CTO diagnosis, the added diagnostic value of UAR and CRP at this stage remains uncertain. As an exploratory study, our findings demonstrate for the first time that their combination can effectively predict CCC quality, offering a new direction for non-invasive risk assessment. Given the increasing use of coronary CT angiography (CTCA) in the early evaluation of CAD, validating the predictive value of these biomarkers in this setting could enable earlier identification of patients with poor CCC. Integrating serum markers with radiomics and artificial intelligence may further advance the development of a non-invasive, precision assessment system for CCC.

## 5 Conclusion

**Figure 4:**
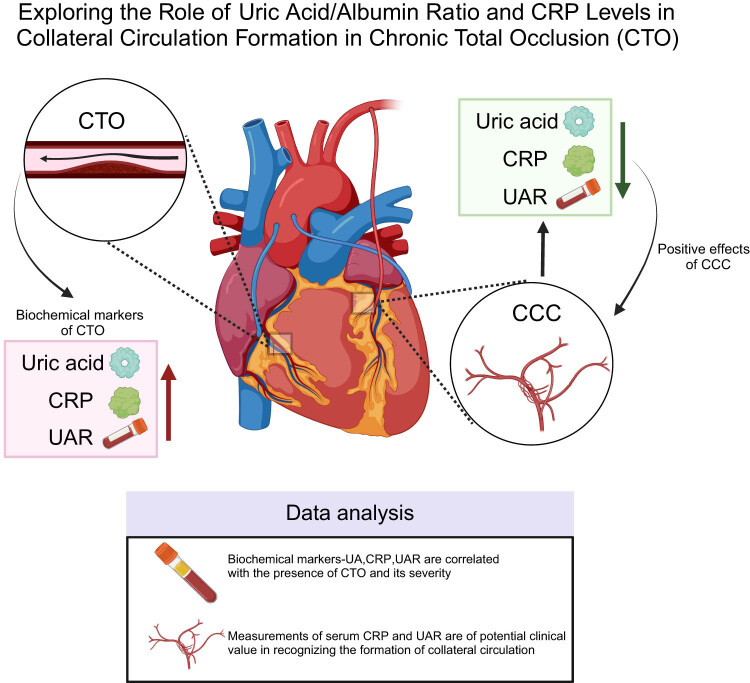
Exploring the role of uric acid/albumin ratio and CRP levels in collateral circulation formation in CTO. Note: CRP: C-reactive protein; CTO: chronic total occlusion; UAR: uric acid-to-albumin ratio; and CCC: coronary collateral circulation.

This study preliminarily reveals the potential value of combining serum CRP and UAR as biomarkers for predicting CCC status in patients with CTO. The findings reveal that elevated CRP and UAR levels are independent risk factors for impaired collateral formation, and their combined assessment can significantly improve diagnostic specificity. These results indicate that CRP and UAR are closely associated with CCC formation but do not fully reflect its complexity, suggesting that collateral formation is a multifactorial process (Figure 4). This study provides a foundation for integrating these biomarkers into clinical practice to better assess cardiovascular risk and prognosis in CTO patients. Although CCC is currently assessed by invasive angiography, future validation of CRP and UAR in non-invasive settings such as CTCA may enable early identification of patients with poor CCC, supporting risk stratification and personalized treatment. Larger, multi-center studies are needed to confirm these findings and further explore the mechanisms underlying CCC development.

## References

[1] Huang F. Is a previously or currently reduced testosterone level in male patients with type 2 diabetes mellitus a risk factor for the development of coronary artery disease? a systematic review and meta-analysis. Diabetes Ther. 2018;9:1061–72. 10.1007/s13300-018-0415-3 PMC5984918 29619752

[2] Lawton JS, Tamis-Holland JE, Bangalore S, Bates ER, Beckie TM, Bischoff JM, et al. 2021 ACC/AHA/SCAI guideline for coronary artery revascularization. J Am Coll Cardiol. 2022;79:e21–e129. 10.1016/j.jacc.2021.09.006 34895950

[3] Golub IS, Termeie OG, Kristo S, Schroeder LP, Lakshmanan S, Shafter AM, et al. Major global coronary artery calcium guidelines. JACC: Cardiovasc Imaging. 2023;16:98–117. 10.1016/j.jcmg.2022.06.018 36599573

[4] Jankowski J, Floege J, Fliser D, Böhm M, Marx N. Cardiovascular disease in chronic kidney disease. Circulation. 2021;143:1157–72. 10.1161/CIRCULATIONAHA.120.050686 PMC7969169 33720773

[5] Marnell CS, Bick A, Natarajan P. Clonal hematopoiesis of indeterminate potential (CHIP): Linking somatic mutations, hematopoiesis, chronic inflammation and cardiovascular disease. J Mol Cell Cardiol. 2021;161:98–105. 10.1016/j.yjmcc.2021.07.004 PMC8629838 34298011

[6] Zuo X, Li X, Tang K, Zhao R, Wu M, Wang Y, et al. Sarcopenia and cardiovascular diseases: A systematic review and meta‐analysis. J Cachexia Sarcopenia Muscle. 2023;14:1183–98. 10.1002/jcsm.13221 PMC10235887 37002802

[7] Stone PH, Libby P, Boden WE. Fundamental pathobiology of coronary atherosclerosis and clinical implications for chronic ischemic heart disease management – the plaque hypothesis. JAMA Cardiol. 2023;8:192. 10.1001/jamacardio.2022.3926 PMC11016334 36515941

[8] Cury RC, Leipsic J, Abbara S, Achenbach S, Berman D, Bittencourt M, et al. CAD-RADSTM 2.0–2022 coronary artery disease-reporting and data system. J Cardiovasc Comput Tomogr. 2022;16:536–57. 10.1016/j.jcct.2022.07.002 35864070

[9] Onnis C, Virmani R, Kawai K, Nardi V, Lerman A, Cademartiri F, et al. Coronary artery calcification: current concepts and clinical implications. Circulation. 2024;149:251–66. 10.1161/CIRCULATIONAHA.123.065657 PMC10794033 38227718

[10] Vaidya Y, Cavanaugh SM, Dhamoon AS. Myocardial stunning and hibernation. In: StatPearls. Treasure Island (FL): StatPearls Publishing; 2025. 30725711

[11] Iftikhar SF, Bishop MA, Hu P. Complex coronary artery lesions. In: StatPearls. Treasure Island (FL): StatPearls Publishing; 2024. 30969721

[12] Leite L, Campos G, Silva R, Jorge E, Oliveira-Santos M, Gomes A, et al. The association of collaterals with myocardial ischemia and viability in chronic total occlusions. Int J Cardiovasc Imaging. 2022;39:843–51. 10.1007/s10554-022-02772-z 36494504

[13] Fagu A, Berger T, Pingpoh C, Kondov S, Kreibich M, Minners J, et al. In-hospital outcomes following surgical revascularization of chronic total coronary occlusions. Medicina. 2023;59:1967. 10.3390/medicina59111967 PMC10673513 38004016

[14] Sanchez-Jimenez E, El-Mokdad R, Chaddad R, Cortese B. Drug-coated balloon for the management of coronary chronic total occlusions. Rev Cardiovasc Med. 2022;23(2):42. 10.31083/j.rcm2302042. 35229533

[15] Allana SS, Kostantinis S, Rempakos A, Simsek B, Karacsonyi J, Alexandrou M, et al. The retrograde approach to chronic total occlusion percutaneous coronary interventions. JACC: Cardiovasc Interventions. 2023;16:2748–62. 10.1016/j.jcin.2023.08.031 38030360

[16] Bhasin D, Shrimanth YS, Sharma YP, Panda P. Vieussens’ arterial ring. J Invasive Cardiol. 2022;34:E343–4. 10.25270/jic/21.00390 35366229

[17] Nepal S, Annamaraju P. Coronary arteriovenous fistula. In: StatPearls. Treasure Island (FL): StatPearls Publishing; 2022. 32119505

[18] Achim A, Johnson NP, Liblik K, Burckhardt A, Krivoshei L, Leibundgut G. Coronary steal: how many thieves are out there? Eur Heart J. 2023;44:2805–14. 10.1093/eurheartj/ehad327 37264699

[19] Zhou H, Huang YS, Zhao Y-T, Zhang C-H, Wang H, Yang X-Y, et al. Clinical and electrocardiographic features in acute total left main coronary artery occlusion without collateral circulation. J Electrocardiol. 2023;76:79–84. 10.1016/j.jelectrocard.2022.11.005 36512934

[20] Calvão J, Braga M, Brandão M, Campinas A, Alexandre A, Amador A, et al. Acute total occlusion of the unprotected left main coronary artery: Patient characteristics and outcomes. Rev Port de Cardiol. 2023;42:723–9. 10.1016/j.repc.2022.11.007 37094728

[21] Alam MK, Alfawzan AA, Srivastava KC, Shrivastava D, Ganji KK, Manay SM. Craniofacial morphology in Apert syndrome: a systematic review and meta-analysis. Sci Rep. 2022;12(1):5708. 10.1038/s41598-022-09764-y. PMC8983770 35383244

[22] Kim Y. The association between red, processed and white meat consumption and risk of pancreatic cancer: a meta-analysis of prospective cohort studies. Cancer Causes Control. 2023;34:569–81. 10.1007/s10552-023-01698-8 37071321

[23] Hao X, Wang S, Jiang C, Zhang J, Fan Y, Pang J, et al. The relation between plasma miR-126 levels and cerebral collateral circulation in patients with intracranial arterial stenosis. Neurol Neurochir Pol. 2021;55:281–8. 10.5603/PJNNS.a2021.0019 33559872

[24] Kang MS, Kim SY, Park SW, Byon IS, Kwon HJ. Association between capillary congestion and macular edema recurrence in chronic branch retinal vein occlusion through quantitative analysis of OCT angiography. Sci Rep. 2021;11(1):19886. 10.1038/s41598-021-99429-z. PMC8494742 34615979

[25] Li Z, Kadian S, Mishra RK, Huang T, Zhou C, Liu S, et al. Electrochemical detection of cholesterol in human biofluid using microneedle sensor. J Mater Chem B. 2023;11:6075–81. 10.1039/d2tb02142k 37254923

[26] Afrose D, Chen H, Ranashinghe A, Liu C, Henessy A, Hansbro PM, et al. The diagnostic potential of oxidative stress biomarkers for preeclampsia: systematic review and meta-analysis. Biol Sex Differ. 2022;13(1):26. 10.1186/s13293-022-00436-0. PMC9167545 35658944

[27] Du K, Zhou Q, Wang Z, Mo C, Dong W, Wei N, et al. Polydatin ameliorates inflammation and oxidative stress associated with MSU-induced gouty arthritis in mice by regulating PPAR-γ and ferritin activation. Life Sci. 2023;326:121766. 10.1016/j.lfs.2023.121766 37209866

[28] Zhou Y, Chen M, Zheng J, Shui X, He Y, Luo H, et al. Insights into the relationship between serum uric acid and pulmonary hypertension (Review). Mol Med Rep. 2023;29(1):10. 10.3892/mmr.2023.13133. PMC10704563 37997855

[29] Mizuno N, Ioka T, Ogawa G, Nakamura S, Hiraoka N, Ito Y, et al. Effect of systemic inflammatory response on induction chemotherapy followed by chemoradiotherapy for locally advanced pancreatic cancer: an exploratory subgroup analysis on systemic inflammatory response in JCOG1106. Jpn J Clin Oncol. 2023;53:704–13. 10.1093/jjco/hyad044 PMC10390851 37248668

[30] Gyawali P, Shrestha H, Pant V, Risal P, Gautam S. C-reactive protein to albumin ratio among patients admitted to intensive care unit of a tertiary care hospital: a descriptive crosssectional study. J Nepal Med Assoc. 2021;59:1247–51. 10.31729/jnma.7047 PMC9200041 35199783

[31] Duran M, Ornek E, Murat SN, Turfan M, Vatankulu MA, Ocak A, et al. High levels of serum uric acid impair development of coronary collaterals in patients with acute coronary syndrome. Angiology. 2011;63:472–5. 10.1177/0003319711422433 21948975

[32] Uysal OK, Sahin DY, Duran M, Turkoglu C, Yıldırım A, Elbasan Z, et al. Association between uric acid and coronary collateral circulation in patients with stable coronary artery disease. Angiology. 2013;65:227–31. 10.1177/0003319713500706 23966572

[33] Toprak K, Yılmaz R, Kaplangoray M, Memioğlu T, İnanır M, Akyol S, et al. Comparison of the effect of uric acid/albumin ratio on coronary colleteral circulation with other inflammation-based markers in stable coronary artery disease patients. Perfusion. 2023;39:1440–52. 10.1177/02676591231202105 37674333

[34] Lin G, Li J, Chen K, Wang A, Guo C. Circ_0000854 regulates the progression of hepatocellular carcinoma through miR-1294/IRGQ axis. Clin Immunol. 2022;238:109007. 10.1016/j.clim.2022.109007 35417749

[35] Chen S, Li L, Wu Z, Liu Y, Li F, Huang K, et al. SerpinG1: A novel biomarker associated with poor coronary collateral in patients with stable coronary disease and chronic total occlusion. JAHA. 2022;11(24):e027614. 10.1161/jaha.122.027614. PMC9798810 36515245

[36] Li X, Zhu J, Zhong Y, Liu C, Yao M, Sun Y, et al. Targeting long noncoding RNA-AQP4-AS1 for the treatment of retinal neurovascular dysfunction in diabetes mellitus. eBioMedicine. 2022;77:103857. 10.1016/j.ebiom.2022.103857 PMC8850682 35172268

[37] Şaylık F, Çınar T, Selçuk M, Tanboğa İH. A Relação entre a Relação Ácido Úrico/Albumina e a Espessura Média-Intimal da Carótida em Pacientes com Hipertensão. Arq Bras Cardiol. 2023;120(5):e20220819. 10.36660/abc.20220819. PMC10124582 37098960

[38] Selçuk M, Çınar T, Şaylık F, Akbulut T, Asal S, Çiçek V, et al. Predictive value of uric acid/albumin ratio for the prediction of new-onset atrial fibrillation in patients with ST-elevation myocardial Infarction. Rev Invest Clin. 2022;74:156–64. 10.24875/RIC.22000072 35797660

[39] Del Pinto R, Viazzi F, Pontremoli R, Ferri C, Carubbi F, Russo E. The URRAH study. Panminerva Med. 2021;63(4):416–23. 10.23736/s0031-0808.21.04357-3. 33765764

[40] Burnier M. Gout and hyperuricaemia: modifiable cardiovascular risk factors? Front Cardiovasc Med. 2023;10:1190069. 10.3389/fcvm.2023.1190069. PMC10248051 37304945

[41] Ridker PM, Bhatt DL, Pradhan AD, Glynn RJ, MacFadyen JG, Nissen SE. Inflammation and cholesterol as predictors of cardiovascular events among patients receiving statin therapy: a collaborative analysis of three randomised trials. Lancet. 2023;401:1293–301. 10.1016/S0140-6736(23)00215-5 36893777

[42] Pope JE, Choy EH. C-reactive protein and implications in rheumatoid arthritis and associated comorbidities. SemArthritis Rheumatism. 2021;51:219–29. 10.1016/j.semarthrit.2020.11.005 33385862

[43] Manolis AA, Manolis TA, Melita H, Mikhailidis DP, Manolis AS. Low serum albumin: A neglected predictor in patients with cardiovascular disease. Eur J Intern Med. 2022;102:24–39. 10.1016/j.ejim.2022.05.004 35537999

[44] Suneson K, Lindahl J, Chamli Hårsmar S, Söderberg G, Lindqvist D. Inflammatory Depression—Mechanisms and Non-Pharmacological Interventions. IJMS. 2021;22:1640. 10.3390/ijms22041640 PMC7915869 33561973

[45] Dix C, Zeller J, Stevens H, Eisenhardt SU, Shing KSCT, Nero TL, et al. C-reactive protein, immunothrombosis and venous thromboembolism. Front Immunol. 2022;13:1002652. 10.3389/fimmu.2022.1002652. PMC9513482 36177015

[46] Liu X, Tong X, Zou Y, Lin X, Zhao H, Tian L, et al. Mendelian randomization analyses support causal relationships between blood metabolites and the gut microbiome. Nat Genet. 2022;54:52–61. 10.1038/s41588-021-00968-y 34980918

[47] Li B, Chen L, Hu X, Tan T, Yang J, Bao W, et al. Association of Serum uric acid with all-cause and cardiovascular mortality in diabetes. Diabetes Care. 2022;46:425–33. 10.2337/dc22-1339 36490263

[48] Yanai H, Adachi H, Hakoshima M, Katsuyama H. Molecular biological and clinical understanding of the pathophysiology and treatments of hyperuricemia and its association with metabolic syndrome, cardiovascular diseases and chronic kidney disease. IJMS. 2021;22:9221. 10.3390/ijms22179221 PMC8431537 34502127

[49] Nacar AB, Erayman A, Kurt M, Buyukkaya E, Karakaş MF, Akcay AB, et al. The relationship between coronary collateral circulation and neutrophil/lymphocyte ratio in patients with coronary chronic total occlusion. Med Princ Pract. 2014;24:65–9. 10.1159/000365734 PMC5588179 25342010

[50] Wu H, Chiou J. Potential benefits of probiotics and prebiotics for coronary heart disease and stroke. Nutrients. 2021;13:2878. 10.3390/nu13082878 PMC8401746 34445037

[51] Nelson K, Fuster V, Ridker PM. Low-dose colchicine for secondary prevention of coronary artery disease. J Am Coll Cardiol. 2023;82:648–60. 10.1016/j.jacc.2023.05.055 37558377

[52] Ballantyne CM, Bays H, Catapano AL, Goldberg A, Ray KK, Saseen JJ. Role of bempedoic acid in clinical practice. Cardiovasc Drugs Ther. 2021;35:853–64. 10.1007/s10557-021-07147-5 PMC8266788 33818688

[53] Levy‐Hirsch L. Transferable skills from ECC career led to managing director role in startup business. Vet Rec. 2023;193(4):ii–iii. 10.1002/vetr.3402. 37594821

[54] Li S, Chen H, Zhou L, Cui H, Liang S, Li H. The uric acid to albumin ratio: a novel predictor of long-term cardiac mortality in patients with unstable angina pectoris after percutaneous coronary intervention. ScJ Clin Lab Invest. 2022;82:304–10. 10.1080/00365513.2022.2084698 35675042

[55] Sultana S, Prakash VR, Karthikeyan A, Kulkarni A, Shaikh MA, GC S. Evaluation of uric acid to albumin ratio as a marker of coronary artery disease severity in acute coronary syndrome: a cross-sectional study. Cureus. 2023;15(11):e49454. 10.7759/cureus.49454. PMC10751248 38152782

[56] Bakhshi H, Michelhaugh SA, Bruce SA, Seliger SL, Qian X, Ambale Venkatesh B, et al. Association between proteomic biomarkers and myocardial fibrosis measured by MRI: the multi-ethnic study of atherosclerosis. eBioMedicine. 2023;90:104490. 10.1016/j.ebiom.2023.104490 PMC10006438 36857966

